# A one-step, tunable method of selective reactive sputter deposition as a wrinkling approach for silver/polydimethylsiloxane for electrically conductive pliable surfaces

**DOI:** 10.1038/s41378-022-00420-z

**Published:** 2022-08-08

**Authors:** Joel Y. Y. Loh, Ali Zeineddine, Moein Shayegannia, Robyn McNeil, Liam McRae, Nazir P. Kherani

**Affiliations:** 1 Department of Electrical and Computing Engineering, University of Toronto, Toronto, ON M5S 3G4 Canada; 2 Department of Material Science and Engineering, University of Toronto, Toronto, ON M5S 3E4 Canada

**Keywords:** Structural properties, Biosensors

## Abstract

The wrinkle period and morphology of a metal thin film on an elastic substrate is typically controlled by modifying the substrate before carrying out additional metal deposition steps. Herein, we show that a simultaneously selective and reactive sputtering plasma that modifies the surface of a polydimethylsiloxane (PDMS) substrate while not reacting with the metal during the deposition process decreases the wrinkle wavelength and induces additional wrinkling components and features such as ripples or folds. The selective reaction of the nitrogen plasma with PDMS functionalizes the siloxane surface into silicon oxynitride. This hardens the immediate surface of PDMS, with a quadratic increase in the Young’s modulus as a function of the sputtering flow ratio. The increase in the critical strain mismatch and the corresponding presence of folds in the nitrogen-modified wrinkled silver film form a suitable plasmonic platform for surface-enhanced Raman spectroscopy (SERS), yielding an enhancement factor of 4.8 × 10^5^ for detecting lipids. This enhancement is linked to the emergence of electromagnetic hotspots from surface plasmon polariton coupling between the folds/wrinkles, which in turn enables the detection of low concentrations of organics using SERS. Furthermore, when strained, the nitrogen-modified wrinkles enhance electrical conductivity by a factor of 12 compared with unmodified films. Finally, the optical properties of the substrate can be tuned by altering the N_2_ content. The simple addition of nonreactive nitrogen to silver sputtering enables simultaneous PDMS hardening and growth of the silver film and together provide a new avenue for tuning wrinkling parameters and enhancing the electrical conductivity of pliable surfaces.

## Graphical abstract

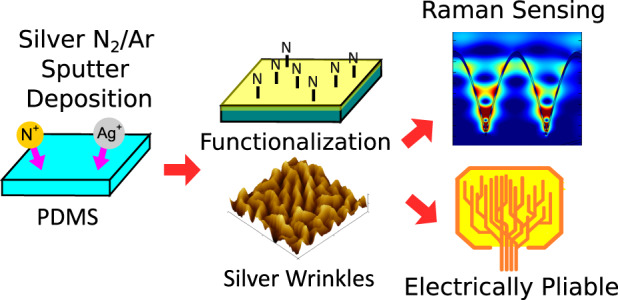

## Introduction

Surface wrinkles can be of benefit to various applications, such as flexible electronics, plasmonic sensing, and tribology-related devices. The implementation of flexible electronics in artificial skin^1–3^ and biosensing^4^ applications requires that thin metal film electrodes undergo a strain of more than a factor of two^5^ without significant degradation in their electrical conductivity. Periodic metallic nanostructures on flexible polydimethylsiloxane (PDMS) substrates have been demonstrated to have tunable plasmonic resonances with high sensitivity^6–8^, which serves as a cost-effective method for hyperspectral sensing^9,10^. For touch-sensing applications such as tactile robotic fingers^11–13^, the increase in the friction coefficient that can be achieved from nano- and microwrinkles enables the extraction of textural information. Hence, a facile method for the formation and control of wrinkles on a metallic film is deemed to be of significant benefit to these applications^14^.

**Fig. 1:**
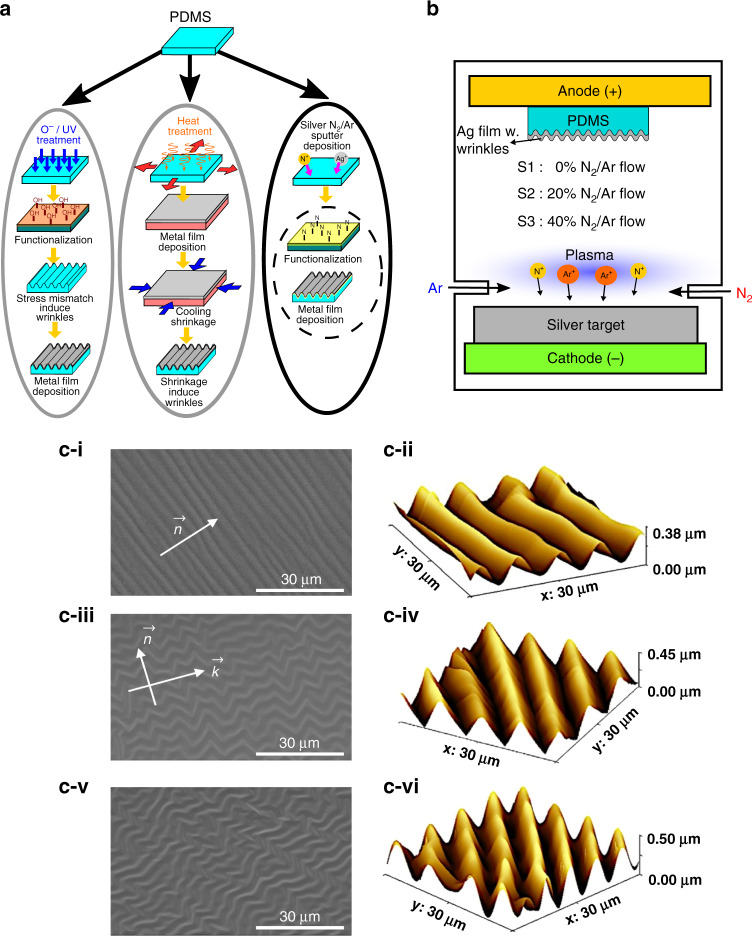
Simultaneous non-reactive and reactive sputter deposition technique for wrinkled Ag/PDMS surfaces **a** Illustration of common wrinkle-inducing processes compared with the nitrogen/argon sputtering of silver onto PDMS: oxygen/UV plasma treatment that modifies a PDMS surface to induce strain mismatch, and heat-deposition-cooling treatment that uses the different shrinking behaviors of metal and PDMS to generate wrinkles. These processes require several steps, whereas nitrogen/argon sputtering is a facile, one-step method, wherein the surface functionalization and metal deposition occur concurrently. **b** Illustration of the sputtering apparatus, where a N^+^/Ar^+^ plasma sheath is generated from N_2_ and argon flow. The plasma sheath of N^+^ and Ar^+^ ions bombard the Ag target to form Ag^+^ ions, residual N^+^ and Ar^+^ ions that travel toward the substrate. The high-kinetic-energy process of plasma-induced “evaporation” can incorporate N^+^ ions within the growing silver film. **c** Morphologies of wrinkled Ag films S1, S2 and S3. (**c-i, c-iii, c-v**) Scanning electron microscopy (SEM) images of wrinkled Ag films S1, S2, and S3. (**c-ii**, **c-iv**, **c-vi**) Surface morphology of S1, S2, and S3 as characterized by atomic force microscopy (AFM).

Typically, the surface of PDMS is modified through thermal expansion and metal evaporation^15^, where the thermal coefficient mismatch induces a compressive stress that is relieved by the formation of wrinkles in the same direction as the applied stress^16,17^. Wrinkles can also be formed using UV radiation damage or by UV lithography-controlled patterning^18–20^, by focused ion beam treatment^21,22^, or by oxygen plasma treatment on prestrained or otherwise prepared PDMS^23–26^. Oxygen plasma treatments involve additional polymer crosslinking and require the methyl surface groups to be replaced with oxygen^27^ to create a stiff SiO_x_ layer that interacts with the underlying strain in the bulk material to form a variety of wave-like patterns or forms alternatively crosslinking modifications through the use of various mixtures of curing and epoxy agents^28,29^ to generate surface morphological wrinkles (Fig. 1a). Recently, it has been shown^30^ that a single-step fabrication process can generate wrinkles; however, this technique relies on resists containing alkyl groups and requires high temperatures for resist “curing” (>150 °C). This technique also requires plasma treatment using a corrosive agent (fluorine) via hexafluoride gas (SF_6_). In general, the aforementioned treatments significantly change the optical, hydrophobic or mechanical properties of PDMS, which can limit the maximum strain of the films before cracking. This has been identified through the formation of silicon oxide-rich PDMS surfaces of varying thicknesses^31^ during oxygen plasma treatment, which can limit exposure time and decrease the degree of controllability. Furthermore, the internal stress of the deposited metal film is dominated by the properties of treated surface, which can be undesirable for the quality of the metallic material itself. Finally, most of the aforementioned treatments of PDMS precede metal deposition, which adds to the complexity and cost of creating very large areas of wrinkled films. A simpler, more cost-effective, and safer one-step method that uses sputter deposition to control the wrinkles over a large area of a metal film can address many of these concerns.

We propose an alternative approach that does not directly modify the PDMS substrate before metal deposition but instead modifies the PDMS surface *en passant* through the growth process during metal deposition on PDMS. While sputtering processes involve the formation of a plasma sheath that bombards the target material with high-kinetic-energy ions (typically Ar^+^), the substrates are generally kept at a distance from the plasma such that the direct plasma interaction is minimized. We show that by introducing nitrogen (N_2_) during the sputtering of silver (Ag) onto an unstrained and unmodified PDMS substrate, the effective mechanical stiffness of a Ag/PDMS substrate can be changed, which induces a variety of controllable morphological changes. The set of wrinkle patterns that emerge from sputtering with various N_2_/Ar ratios can be used as a facile means for producing affordable surface-enhanced Raman spectroscopy (SERS) sensing substrates, flexible electronic skins and pliable conducting surfaces for other applications. In fact, in prior work^32^^,^ we showed that N_2_-sputtered Ag can induce Ag nanoparticle diffusion into an adjacent dielectric substrate, through which both localized and surface plasmon resonances can interact.

In this investigation, a PDMS substrate was conventionally fabricated with no pre- or postmodification treatments. A 5 nm chromium adhesion layer was first evaporated onto the PDMS film, followed by RF sputter deposition of a 25 nm thick silver film (Fig. 1b) under N_2_/Ar flow ratios of 0% (S1), 20% (S2) and 40% (S3) N_2_/Ar. To understand how nitrogen-incorporated Ag deposition modifies the stress mismatch between PDMS and the Ag film, we conducted time-of-flight secondary ion mass spectrometry (ToF-SIMS) and X-ray photoelectron spectroscopy (XPS) to obtain the etched depth profiles of samples with different N_2_/Ar flow ratios on PDMS and of flat planar silicon substrates. To eliminate the possibility of thermally induced effects, we further investigated the deposition of Ag at a high PDMS substrate temperature. The effect of the deposition parameters on the Young’s modulus of PDMS was determined analytically, and we showed that the trends in the critical stress and Young’s modulus as a function of the N_2_/Ar flow ratio can be validated by the predicted and experimental values of the wrinkle wavelengths. To determine the effectiveness of N_2_-modified Ag/PDMS as a low-cost SERS substrate, we conducted lipid sensitivity measurements, and to test for improvements in the N_2_-modified Ag/PDMS substrates for the field of flexible electronics, we also carried out electronic characterization under uniaxial straining using a custom-designed strain-application device.

## Results and discussion

Figure 1c–i shows that there is one dominant surface morphology that seemingly evolves in control sample S1, which was produced with a 0% N_2_/Ar ratio. S1 exhibits a homogeneous 1D sinusoidal wrinkling pattern along the propagation direction of the wrinkles, $\vec n$, as seen in the inset. Utilizing the AFM image in Fig. 1c-ii, the average wavelength of the wrinkles is calculated via fast Fourier transform (FFT) to be ~3.2–3.5 μm, with an average wrinkle amplitude of 0.35 μm. We note, however, that due to artifacts arising from probe tip-sample convolution, the wrinkle periods as determined by AFM may be larger than those shown in the SEM images by up to 20%. According to linear^33^ and nonlinear^34^ stability analysis, a metal-polymer bilayer structure of two different stiffness moduli buckles from the tendency to minimize the compression-induced strain energy while increasing the bending energy of the metal film. For 1D wrinkles, the stiffness along the direction perpendicular to the wrinkles is near zero, while the film remains elastic along the wrinkle direction. A sinusoidal wrinkle thus arises when a PDMS substrate is uniaxially prestrained before plasma treatment or during metal deposition. Compared with the commonly employed metal evaporation technique, the sputtering process results in the deposition of atoms with higher kinetic energy; thus, the surface diffusion of metal adatoms likely plays a role in the strain energy of the film. For Ag sputter deposition, unconnected atomic islands grow both laterally and vertically at thicknesses less than 10 nm, and as the islands meet to form grain boundaries, the initial tensile stress is replaced by a compressive stress as a continuous film is formed at thicknesses greater than 10 nm. The compressive stress increases with the density of grain boundaries, which is further associated with high adatom mobility. This implies that the Ag thin film formed by sputter deposition must form wrinkles of high bending energy to relax the compressive stresses imposed by the underlying PDMS substrate and its formation.

An examination of sample S2 shows that the introduction of N_2_ during deposition induces various morphologies that seemingly evolved into a 2D herringbone pattern resembling a series of zigzags, which is a buckling mode that is different from the elementary 1D sinusoidal pattern. The herringbone pattern has been shown to be highly versatile in mitigating biaxial strain, where the alternating directions of the zigzagging pattern minimize the overall in-plane film stress in all directions. Figure 1c-ii shows that the stresses are not only higher in the $\vec n$ direction but also arise in the $\vec k$ direction, signifying biaxial strain. In our films, the short wavelength is approximately λ_0_ = 2.72 µm, and the intermediate wavelength is λ_1_ = 3.00 µm. FFT calculations show that the average wavelength is approximately 2.80 µm. The inclination angle θ spans a range of values. While the herringbone patterns are found to be energetically invariant with the range of inclination angles, there are high bending energies at localities where the inclination angles are small. In sample S3 (Fig. 1c-iii), the increased N_2_ content during the sputtering process induces ridge-like deformations at specific points in the herringbone pattern, occurring relatively perpendicular to the herringbone wrinkles and extending for long distances of up to 33 µm. These points seemingly have short wavelengths of λ = 2.3 µm and small inclination angles. In contrast, the rest of the herringbone pattern exhibits very broad inclination angles to the extent that they almost seem to be straight wrinkles. In S3, ridge deformations occur where there are sharp inclinations of the herringbone wrinkles. These ridge deformations are likely associated with plasticity effects. Yin et al. conducted a finite element analysis on plastoelastic films, where the plastic behavior is associated with flow theory. It assumes that the critical yield torsional stress for the plastic regime is smaller than the critical yield principal stress according to the von Mises criterion of $\tau _Y = Y/\sqrt 3$, where *τ* is the torsional stress and *Y* is the yield point under principal stress. $k = \sigma _y\left( {1 - \nu _f} \right)/E_f$ is the normalized yield stress as well as the biaxial yield strain, where $\sigma _y$ is the plastic yield stress. Accordingly, if the yield stress *k* is greater than the critical stress value, then the film first elastically deforms into herringbones, with plastic deformation occurring afterward to mitigate residual stresses. Since the ridge deformations are nearly perpendicular to the near-linear wrinkles, the elastic wrinkles may mitigate the stresses along one axis, while residual stresses perpendicular to this axis are relieved by plastic deformation.

**Fig. 2:**
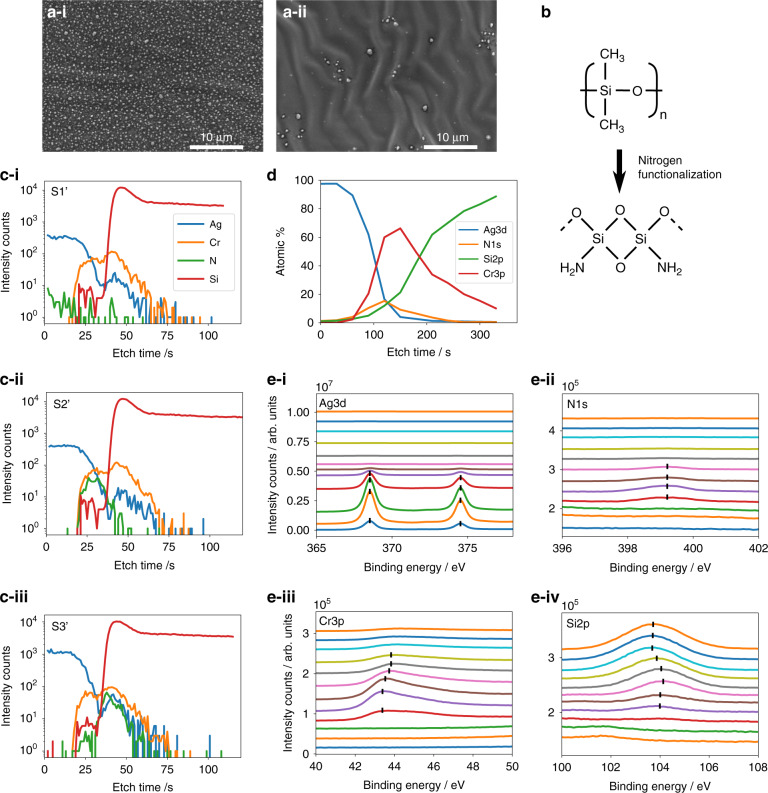
SEM images and ToF-SIMS and XPS profiles. **a-i, a-ii** SEM images of S4 and S5. The PDMS substrate was heated to 50 °C while the Ag film was deposited with 0% and 40% N_2_ flow ratios, respectively. **b** Schematic illustrations of the polymerization of PDMS by N_2_ plasma treatment. **c** ToF-SIMS measurement data for sputtered Ag/Cr on Si: S1’, S2’, and S3’ for 0% N_2_ (**i**), 20% N_2_ (**ii**), and 40% N_2_ (**iii**) flow ratios. **d** Atomic percentage profiles as derived from the XPS spectrum. **e** X-ray spectra of Ag 3d (**i**), N 1 s (**ii**), Si 2p (**iii**), and Cr3p (**iv**) for Ag/Cr/PDMS with a 40% N_2_ flow ratio.

To rule out plasma heating effects, a set of samples were fabricated under thermal heating conditions. The samples, denoted S4 and S5, associated with 0% N_2_ and 40% N_2_ flow ratios, respectively, were heated to 50 °C prior to and during the deposition of the Ag film. Figure 2a-i shows the surface morphology of S4 (0% N_2_ at 50 °C), where two major differences can be seen: suppression of wrinkle continuity and formation of silver nanoparticles. The suppression of the wrinkles and formation of particles can be attributed to the dominating effect of Ag-island growth and the suppression of lateral island growth. For S5 (Fig. 2a-ii), the introduction of N_2_ gas at a ratio of 40% leads to a behavior similar to that observed in S4. The wrinkle wavelengths are still of the same order of magnitude (2–3 µm), but the amplitudes and folds are somewhat suppressed. The formation of nanoparticles can also be seen to occur minimally throughout the sample, indicating that the introduction of N_2_ promotes the lateral growth of Ag islands. The continuity and homogeneity of the Ag film in S5 is in agreement with studies of rigid and weakly interacting substrates. The introduction of N_2_ gas during Ag sputtering in an Ar-rich environment on flat silicon substrates has been shown to create ultrasmooth films with a roughness of 0.6 nm RMS for 15 nm films^35^, confirmed by a drop in the localized surface plasmon resonance peaks of the films compared with those sputtered without N_2_. In addition to optical modeling, real-time in situ film growth monitoring^36^ revealed that the presence of N_2_ during the sputtering process promotes 2D film growth and smooth film surfaces along with an increase in the continuous layer electrical resistivity. Moreover, ex situ structural analysis^36^ suggests that a N_2_ plasma supports 2D morphologies by suppressing the island coalescence rate during the initial growth stages. Although these studies were conducted on rigid substrates, Fig. 2a confirms that the introduction of N_2_ to the sputtering process results in the preferred flat morphology. This can also be a direct cause of the spontaneous biaxial stress emerging on the surface, manifesting as higher-order wrinkles and folds when a continuous film is formed in the presence of the N_2_ plasma. Additionally, it has been shown that the buckling of a gold film deposited on a heated PDMS substrate stemmed from a large mismatch in the thermal expansion coefficients between PDMS and Au. Notably, the compressive stresses relieved by buckling resulted in wrinkles with uniform wavelengths in the range of 20–50 µm^37^, 10 times larger than the wrinkles detected in our samples.

Polymerization of the surface by plasma treatments causes the formation of a silica-like layer (Fig. 2b), which perturbs the elastic modulus of the substrate and induces stiffer surface tension with the overlying film. For O_2_ and Ar plasma treatments^38,39^, the incorporation of oxygen-containing groups has been documented, whereas nitrogen and nitrogen-containing moieties were shown to be incorporated after N_2_ and NH_3_ plasma treatment^40,41^. Except for the N_2_ and NH_3_ treatments that resulted in the additional inclusion of nitrogen moieties, the aforementioned plasma treatments resulted in the dehydrogenation of the PDMS surface, with the incorporation of oxygen in place of the displaced hydrogen^42^. To study the chemical influence of N_2_ sputtering, we conducted a series of sputtering depositions on Si substrates under the same conditions as S1-3, where the layered structures of the form Si/Cr (5 nm)/Ag (20 nm) are denoted S1’, S2’ and S3’. The ToF-SIMS data in Fig. 2c show that a detectable percentage of N is also partially incorporated inside the Cr and PDMS surface layers. Since the nominally 5 nm thick Cr layer is highly porous (SI Fig. 2), N^+^ penetration into PDMS is likely not hindered by the Cr layer. For S1’, the N^+^ intensity is negligible, whereas the N^+^ intensity of S’2 and S’3 increases within the multilayered structure to the Cr/Si interface. The absence of detectable N^+^ within Ag implies that residual N_2_ gases are desorbed from the film postsputtering, and the detected N^+^ at the Cr/Si interface is likely chemically incorporated. Accordingly, for an S3 sample, we carried out XPS analysis at various etched depths at approximately 2–3 nm intervals. The emergence of the N 1 s signal coincides with the appearance of Cr 3p and Si 2p peaks, confirming that N is present at the Cr/PDMS interface (Fig. 2d). The chemical effect was determined by examining the binding energies of Ag 3d, N 1 s, Si 2p, and Cr 3p, as shown in Fig. 2e. The Ag 3d peaks are associated with metallic Ag. A negligible amount of a N 1 s signal is present before the Ag/Cr interface, signifying (along with the binding energy of Ag) that the silver film does not react with nitrogen and that no Ag-N compounds form. Figure 2e-iv shows the binding energies of Cr 3p present in S3. At the Ag/Cr interface, the binding energy is 43 eV, in agreement with the documented value^43^ for Cr metal (42.7 ± 0.4 eV). A higher binding energy (44.4 eV) has been shown^44^ to signify the formation of CrN, and this shift can be seen in the vicinity of the Cr/PDMS interface, which is also confirmed by a shift in the Si 2p peak, as discussed later. This signifies that N^+^ ions selectively appear at the Cr/PDMS interface, either via diffusion through the thin Cr film or due to the discontinuity of the Cr film.

The Si 2p spectrum in Fig. 2e-iii shows two distinct peak positions, with an observed increase in the peak binding energy from 101.5 ± 0.4 eV^45^ to 103 eV and 103.5 eV. Previously, PDMS has been shown to undergo polymerization under various plasma treatments, and as such, the Si-2p peak shifts to higher values of 103.2 eV, 103 ± 0.4 eV and 103.5 ± 0.4 eV under Ar, O_2_ and N_2_ plasma treatments, respectively. The Si 2p peak under a N_2_ plasma treatment is also consistent with that of silica. The N_2_ plasma-treated surface has the highest oxygen content, indicating a higher coverage of the generated silica-like layer. In our samples, a shift to a higher binding energy (103.5 eV) is apparent at the Cr/PDMS interface, while a lower shift (103 eV) is indicated at higher etching times. This is a strong indicator that the PDMS samples undergo in situ plasma surface modification simultaneously with Ag film deposition. It has been shown that the diffusion of nitrogen gas in polymers is markedly different than that of heavier molecules;^46,47^ nitrogen gas tends to cluster on the surface with a penetration depth of only a few nanometers in the same time frame that carbon dioxide passes through the polymer bulk. The high incidence of nitrogen ions on the PDMS surface enables diffusion to proceed laterally throughout the interface of the Cr and PDMS. The high coverage of the silica-like layer on the Cr/PDMS interface starts to gradually decrease beyond the surface of the PDMS sample, providing a gradient change in the surface morphology and mechanical properties of the surface. The polymerization of the surface of the PDMS substrate likely forces a local change in the material properties by the generation of a harder surface layer with a higher Young’s modulus. The Young’s modulus mismatch between the substrate and metal film is decreased; hence, the generated wrinkling and folding are expected to decrease. The PDMS substrate was modified during the Ag film growth process and before the onset of film coalescence, and the associated film/PDMS strain mismatch occurred, thus providing a methodology to generate wrinkles and folds during thin film deposition. Plasma pretreated PDMS has been reported to subsequently relax^48^ as the silica layer decays under oxidation in the atmosphere. With our approach, simultaneous Ag deposition hinders the relaxation of PDMS and maximizes the film/PDMS strain mismatch.

To describe surface wrinkling, two approaches have been previously implemented: a force balance approach and an energy balance approach. We consider the force balance approach with a plate (film) on a semi-infinite substrate. The change in Young’s modulus can be calculated for a bilayer structure via the classical equation for the bending of a stiff film:^49^

1 $$\bar E_fI\frac{{d^4z}}{{dx^4}} + F\frac{{d^2z}}{{dx^2}} + kz = 0$$

where $\bar E = E/{{{\mathrm{(}}}}1 - {\upnu}^2{{{\mathrm{\}}} }}$ is the plane strain modulus ($\bar E_f$ and $\bar E_s$ are the moduli for film and substrate, respectively), ν is Poisson’s ratio, *F* is the uniaxially applied force, *I* is the moment of inertia, and *k* is Winkler’s modulus of elastic half-space ($k = \bar E_s\ {\mathrm w}\pi/\lambda$); *z* is the deflection normal to the surface while *x* is parallel to the force. The force balance equation thus provides a relationship between the wrinkle wavelength *λ*, thin film thickness *h*, thin film width *w*, and mechanical properties of the substrate:^50^

2 $${\uplambda}\left( h \right) = 2{\uppi}h\left( {\bar E_f/3\bar E_s} \right)^{\frac{1}{3}}$$

**Fig. 3:**
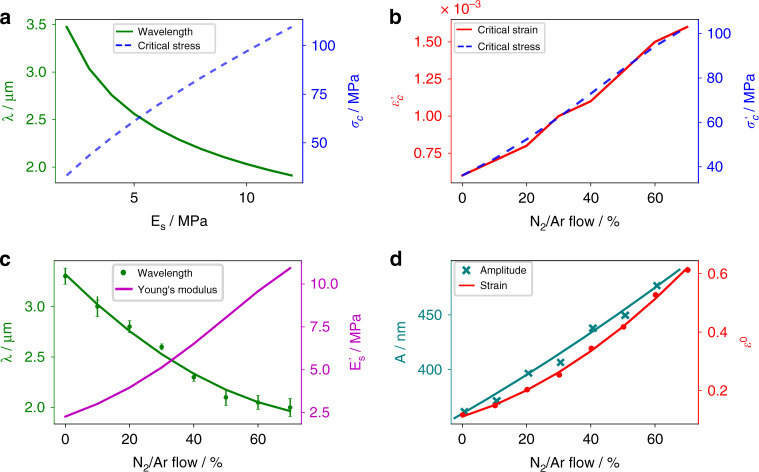
Mechanical properties of Ag/PDMS. **a** Values of the wrinkle wavelength and critical stress as calculated from Eqs. (2) and (3). **b** Critical strain and stress as evaluated from the experimental values of λ. **c** Experimental wrinkle wavelength with corresponding error bars and fit. The evaluated value of the Young’s modulus is also plotted from λ and Eq. (2). **d** Experimental values of the wrinkle amplitudes (*A*) and corresponding strain evaluated by Eq. (4).

Equation (2) provides the basis for surface wrinkling metrology. Using the values for the elastic moduli of thin silver and PDMS (the starting value of ~2.3 MPa pertains to the 10:1 base-to-agent mass ratio), the wrinkle values were plotted (Fig. 3a) as a function of the increasing elastic modulus of PDMS. This relates to the XPS results showing the polymerization of the surface of PDMS and, as such, an increase in the Young’s modulus. The wavelength decreases as the elastic modulus increases in response to the higher strain/stress induced by the “harder” substrate. These values provide an approximate expectation value for the experimental wavelength in relation to the polymerization of the surface of PDMS. The initial calculated wavelength at *E*_*s*_ = 2.3 MPa (representing no change in the elastic modulus of PDMS) is approximately 3.5 μm and decreases to 2 μm at *E*_*s*_ = 11 MPa. These results agree with the values obtained via AFM measurements and FFT calculations of the PDMS/Ag structure under 0–70% N_2_. The experimental values of the wrinkle wavelength (λ’) are plotted in Fig. 3c. As the N_2_ content increases, λ’ decreases, and the value of the substrate elastic modulus quadratically increases. Hence, varying the N_2_/Ar ratio during sputtering provides a direct means for tuning the wavelength of the PDMS/Ag structure with a tunability factor of 50%.

To evaluate the strain produced between the Ag film and the PDMS substrate due to the film/substrate mismatch and polymerization of the substrate, the critical stress must be calculated^49^. Figure 3b and d were plotted using Eqs. (3) and (4).

3 $$\sigma _c = \left( {\frac{9}{{64}}\bar E_f\overline {E_s^2} } \right)^{1/3}$$

4 $$\varepsilon = \varepsilon _c\left[ {\frac{{A^2}}{{h^2}} + 1} \right]$$

where *A* is the amplitude of the wrinkles evaluated via FFT, *h* is the film thickness and ε_c_= σ_c_/$\bar E_f$ is the critical strain where σ_c_ is the critical stress.

The critical strain for polystyrene/PDMS has been shown to be approximately 1%^46^, whereas in our setup, the critical strain $\varepsilon \prime _c$ is 0.06% < $\varepsilon _c$ < 0.2%. This is expected as the Young’s modulus of polystyrene is 3250 MPa, whereas that of silver is approximately 65 GPa for thin films. As shown in Fig. 3d, the amplitude variation with N_2_ content is linear, and as such, the corresponding change in the strain is parabolic. The change in the amplitude of the wrinkles and folds between 0% N_2_ and 70% N_2_ is approximately 30%, allowing for an additional degree of freedom for fabricating self-emerging structures. The aforementioned study not only provides the necessary arguments to explain the process in which the Ag/PDMS structure starts to wrinkle and fold but also provides a recipe for fabricating structures with the desired wavelength, amplitude and strain.

**Fig. 4:**
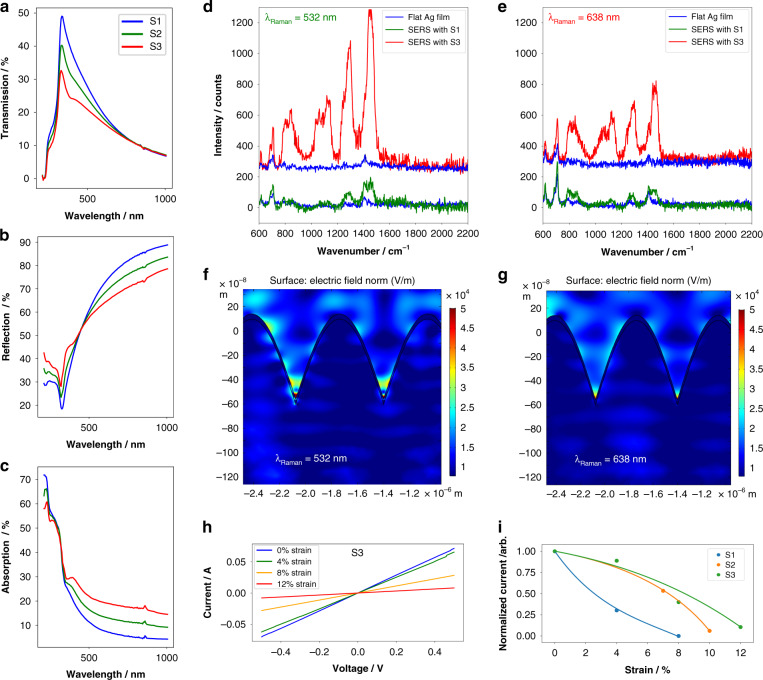
Optical and electrical measurements. **a** UV–Vis transmission. **b** Reflection. **c** Absorption spectra of S1-3 showing greater optical absorption for higher N_2_ content Ag/PDMS. **d**, **e** Spot Raman spectra of lipid-coated S1 and S3 under 532 nm and 638 nm excitation wavelengths. The blue lines are the Raman spectra of the lipids with no SERS on a flat Ag film on silicon substrate. Average electric field intensities at hotspots of sinusoidal wrinkles under (**f**) 532 nm and (**g**) 638 nm excitation. **h** Electrical resistance plots over −0.5 to 0.5 V under 0% to 12% strain. **I** Normalized current profiles of S1-3 under strain. S3 shows the greatest electrical robustness under strain

To study the effects of N_2_ content on optical properties, the total transmittance and reflectance of S1, S2, and S3 were measured via an integrating sphere module using a UV–Vis spectrophotometer from 200 nm to 1000 nm. The optical properties of thin films on glass have been shown to be highly dependent on the surface roughness/smoothness of the thin films. For high surface roughness, localized surface plasmon resonance (LSPR) absorption losses contribute to optical absorption losses^51^. Figure 4a shows a decrease in transmission from 49% for S1 to 32.7% for S3 with a minor blueshift from 335 nm to 330 nm. The reflection spectra in Fig. 4b also follow a similar trend toward a decrease in magnitude from S1 to S3. Reflection magnitude reversal starts at 345 nm, and upon surpassing 450 nm, the S3 reflection becomes lower than that of S1. Figure 4c shows the absorption values of the three samples S1, S2 and S3. As the N_2_ content increases from 0% to 40%, the absorption increases, and the absorption of S3 becomes larger than that of S1 above 320 nm. We noted the formation of local maxima as the N_2_ content increased. For S1, there is no local maxima, but S3 clearly shows one at 400 nm, resembling the LSPR of nanoparticles^51^. The LSPR is believed to arise from the surface morphological changes of the structures where biaxial stresses cause the film to wrinkle and fold in the $\vec n$ and $\vec k$ directions. The position of the LSPR is in agreement with that found in the literature^51^ for small nanoparticles (20 nm). The UV–Vis measurements of S3 show that the optical response of the Ag/PDMS structure can be tuned via the introduction of N_2_ to provide a more absorptive, less reflective substrate. This has major implications, especially for SERS. The increase in absorption in addition to the formation of LSPR provides a low-cost SERS substrate capable of enhancing Raman signals.

The Ag/PDMS substrate can be an effective platform for SERS applications due to its surface roughness and wrinkled structure. To study the functionality of this substrate as a sensing platform, we conducted a series of SERS characterizations using dried phospholipid 1,2-dipalmitoyl-sn-glycero-3-phosphoethanolamine conjugated polyethylene glycol (DPPE-PEG) coated on a freshly fabricated Ag/PDMS substrate. In general, phospholipids form the membrane of living cells and are responsible for cell biosynthesis and immunological recognition. Additionally, PEGylated phospholipids are excellent bilayer and liposome formulations for drug and vaccine delivery. Hence, the detection of DPPE-PEG is helpful for accurate prognoses and advancing therapeutic approaches^52^.

The SERS spectra of DPPE-PEG at 532 nm and 638 nm are shown in Fig. 4d, e for S1 and S3. In both figures, the blue spectra show the Raman spectra of DPPE-PEG on a flat silver film on planar silicon. With an increase in the nitrogen content from 0% to 40% within the substrate, the SERS spectra of the platform at both excitation wavelengths clearly improves. At 532 nm, S3 offers a SERS intensity of 1300 counts at a wavenumber of 1458 cm^−1^, while the same vibrational mode has a Raman count of less than 200 for S1. In other words, increasing the nitrogen content from 0% to 40% improves the SERS intensity by more than 8-fold at 532 nm and approximately by 4-fold at 638 nm. More importantly, at both excitation wavelengths, vibrational modes between 1000 cm^−1^ and 1200 cm^−1^, which correspond to the phosphate group and CO–O–C of the phospholipid, are revealed only when S3 is used as the SERS substrate.

To calculate the enhancement factor, we assume that the phospholipid molecules dry uniformly over the focal diameter of the laser beam on the Ag/PDMS substrate. Hence, we hypothesize that the number of deposited molecules on the SERS platform is proportional to the volume of an inverted cone within the sinusoidal pattern of the wrinkle structure of the substrate. Additionally, the number of molecules on a non-SERS platform is taken to be the focal area of the laser beam when illuminating a flat silver film. As mentioned before and shown in Fig. 1, S1 and S3 have wrinkle amplitudes of 0.35 µm and 0.5 µm and wavelengths of ~3.3 µm and ~2.3 µm, respectively. Since both of these structural wavelengths are larger than the focal diameter of the laser beam (~2 µm), we consider a width of 2 µm for the inverted cone where the molecules are deposited. Hence, the calculated inverted cone volumes for S1 and S3 are 4.4 µm^3^ and 6.28 µm^3^, respectively. Thus, the SERS enhancement factors at the vibrational mode of 1458 cm^−1^ for S1 and S3 are 6.1 × 10^4^ and 4.8 × 10^5^ at 532 nm and are 2.2 × 10^4^ and 2 × 10^5^ at 638 nm, respectively. For these calculations, I_SERS_ was taken from the amplitude of the red or green plots, and I_Raman_ was taken from the amplitude of the blue curve at 1458 cm^−1^.

To understand why the SERS enhancement factor at 532 nm is higher than that at 638 nm, we conducted COMSOL-Multiphysics simulations on a sinusoidal structure resembling the wrinkle patterns on the Ag/PDMS structure (Fig. 4f, g). Observation of these figures at 532 nm reveals a larger volume within the valley of the sinusoid (where most of the lipid molecules are assumed to be deposited), supporting plasmonic hotspots. In contrast, at 638 nm, the plasmonic hotspots are concentrated at the bottom of the valley. Hence, at 532 nm, a larger number of phospholipid molecules are excited; therefore, more photons are Raman-scattered.

The robustness of the wrinkles under mechanical strain is of importance to the viability of flexible electronics. Figure 4h shows that with increasing strain, the electrical conductivity does not significantly decrease until 12% strain, and a nonnegligible current is retained under high strain. This is likely associated with the rope-like ridges observed under high strain (see supplementary information), which may contain significant plastic components along the uniaxial direction. Figure 4i shows the normalized current-strain profiles of S1-3. S1 shows fracturing of the film at 9% strain, which correlates with the negligible current at 8% strain. Both S2 and S3 show similar profiles in terms of extending the mechanical robustness to 10–12%, with high electrical currents seen at lower strains. This enhancement in current is nearly 3.6-fold at a low strain of 4%, showing that a minor modification in sputtering deposition can achieve an enormous benefit for flexible electronics.

## Conclusion

We have demonstrated the use of a new tunable factor for modifying the wrinkle morphology of metal/polymeric films. By utilizing a plasma gas component that does not react with the metal film but simultaneously and reactively functionalizes the polymeric surface, we achieve an approach that reduces the number of fabrication steps to yield a faster single-step process, where the polymer stress relaxation seen postplasma functionalization is eliminated. Through XPS and ToF-SIMS analysis, nitrogen ions generated in a N_2_/Ar plasma mixture interact with the Cr/PDMS interface to induce a hard surface, while Ag remains unreacted. Notably, we have shown that the changes in the Young’s modulus of PDMS can be determined as a function of the N_2_/Ar flow ratio, allowing for high variability in the stress mismatch within a factor of 4. Due to the high mismatch, folds and ripples are observed, along with a decrease in the wrinkle wavelength. These modifications result in significant changes in optical transmittance and IR absorption, with a large enhancement in the sensitivity for organic detection. This paves the way for improved and low-cost SERS substrates. In addition, the electrical conductivity of the N-modified Ag/PDMS is maintained even under strain, with high conductivity values similar to those of unmodified films at up to 5 times higher strains. Thus, these results show that enormous advantages arise from minor adjustments to sputter deposition technology. Furthermore, this approach can be generalized to other gases, metals and polymers to create a variety of wrinkle morphologies optimized for sensing and flexible electronic applications.

## Supplementary information

Supplementary Information for “A One-Step, Tunable Method of Selective Reactive Sputter Deposition as a Wrinkling Approach for Silver/Polydimethylsiloxane for Electrically Conductive Pliable Surfaces”
