# Supplementary material for: A one-step, tunable method of selective reactive sputter deposition as a wrinkling approach for silver/polydimethylsiloxane for electrically conductive pliable surfaces
Source: Microsyst Nanoeng. 2022 Aug 8;8:89. doi: 10.1038/s41378-022-00420-z (PMC9360048; doi:10.1038/s41378-022-00420-z)
Supplement: Supplementary file 1 — Supplementary Information for “A One-Step, Tunable Method of Selective Reactive Sputter Deposition as a Wrinkling Approach for Silver/Polydimethylsiloxane for Electrically Conductive Pliable Surfaces” [file 41378_2022_420_MOESM1_ESM.pdf]

Supplementary Information for
"A One-Step, Tunable Method of Selective Reactive Sputter Deposition as a Wrinkling Approach for Silver/Polydimethylsiloxane for Electrically Conductive Pliable Surfaces."

Joel Y.Y Loh[a, †], Ali Zeineddine[a, †], Moein Shayegannia[a], Robyn McNeil[b], Nazir P. Kherani[a,b,*]

[a] Department of Electrical and Computing Engineering, University of Toronto, Toronto, Ontario M5S 3G4, Canada.

[b] Department of Material Science and Engineering, University of Toronto, Toronto, Ontario M5S 3E4, Canada.

[†] Co-first authors who have contributed equally.

*Corresponding author: nazir.kherani@utoronto.ca

SEM Examination of Ag/PDMS using Custom-designed Strain-application Device.

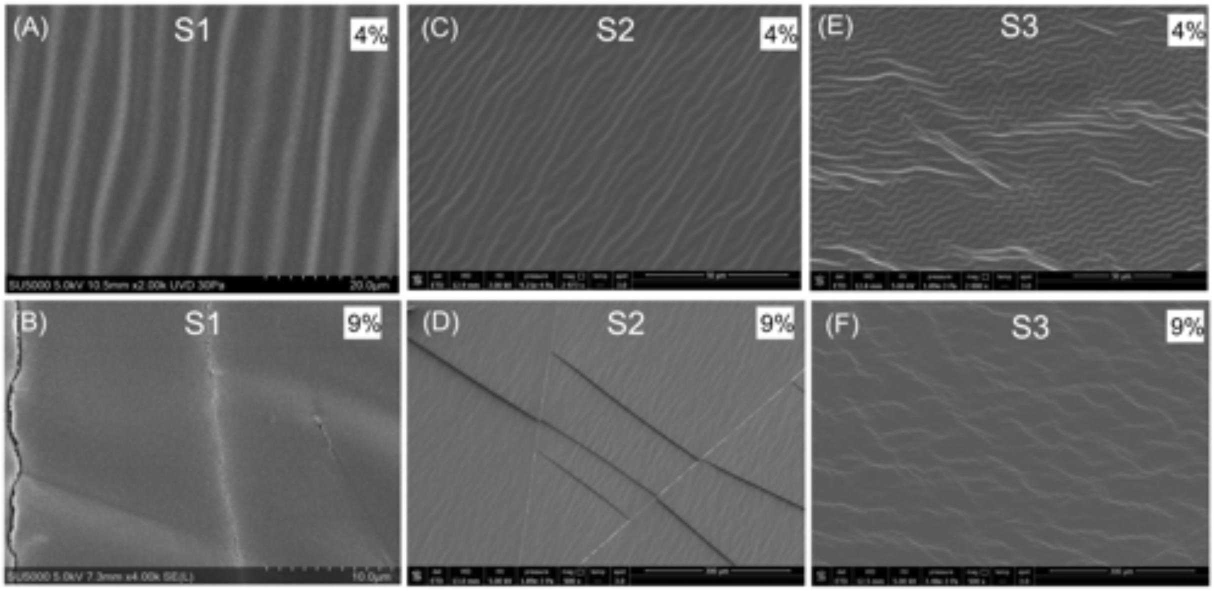

SI Figure 1. In-situ SEM images of S1-S3 under applied strain of 4% and 9%. (**A**) shows that the wavelength of the linear wrinkles of S1 has increased. (**B**) S1 shows near-flattening of wrinkles and cracking failure. (**C**) shows that the herringbone pattern of S2 has straightened into linear wrinkles. (**D**). Terrace like cracks appear perpendicular to the direction of the wrinkles. (**E**) More plastic deformation observed in S2 under 4% strain. (**F**) The plastic deformations result in a diamond-like pattern across the herringbone patterned surface.

The external uni-axial strain induces additional features as seen in **SI Figure 1**. Under a small strain, S2 experiences an increase in wrinkle wavelength while forming near linear wrinkles as the zig-zag herringbone pattern straightens out (**SI Figure 1A**). Under a large strain, cracks up to 20μm wide (**SI Figure 1B**) are seen perpendicular to the linear wrinkles, which denotes failure of the herringbone pattern to accommodate the additional lengthening of the film.

For S3, the herringbone pattern remains while the plastic deformations grow in length (**SI Figure 1E**). Noticeably, under a larger strain a regular pattern of the plastic deformation emerges (**SI Figure 1F**). This diamond-like pattern mixed with a herringbone pattern is also observed in elasto-plastic wrinkling where the ratio of the normalized yield strain over the critical elastic stress is between 1.2 and 2.8 [1]. In the same manner as observed in S3 under strain, it was determined that the herringbone pattern transits to a dominant diamond pattern or a mixed pattern as applied strain is increased. After the diamond pattern emerges to relieve strain, excess strain is subsequently mitigated by greater wrinkle amplitudes and increased number of plastic ridges. No cracking was observed under 9% strain. In all, different sets of patterns evolve under

applied strain based on the varying concentrations of N_2 sputtering, which indicates that the introduction of N_2 causes significant changes to both the substrate and film such that elasto-plastic deformation is enhanced.

<u>SEM Examination of 10 nm Ag film on 5nm Cr Layer on PDMS</u>

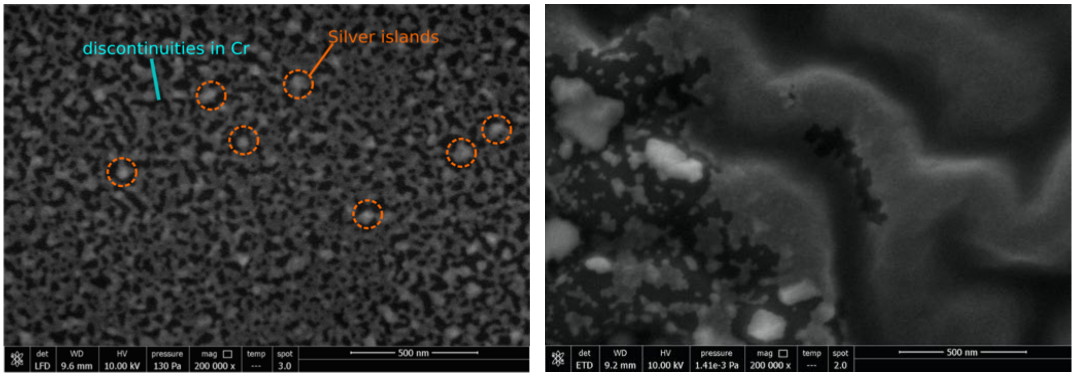

SI Figure 2. Left: 10nm of Ag sputtered with only argon plasma on 5nm of Cr on the PDMS surface. A porous Cr network that exposes the PDMS surface is evident. Right: 10nm of Ag with 65% Ar flow ratio on 5nm Cr on PDMS surface, showing coalescence of Ag nano-islands to form pseudo-herringbone wrinkles.

N^+ diffusion from the deposition of Ag into the PDMS sub-surface can occur via discontinuities in the Cr film. As shown in **SI Figure 2**, where a 5nm Cr film overlaid with a ~10nm thin layer of Ag (0% N_2, 100% Ar flow) film (to prevent charging effects from obscuring the surface yet forms discontinuous Ag islands to expose the underlying Cr), showed that the Cr film is essentially a porous network-like mesh. To prove that chemical hardening of the subsurface beneath the Cr/Ag islands can occur, we deposited 10nm of Ag in a N_2 rich environment (65% N_2, 35% Ar). Instead of undefined nano-islands of silver, silver is shown to take on the form of herringbone wrinkles, where silver has greater coalescence.

UV-Vis Optical Changes using Custom-designed Strain-application Device.

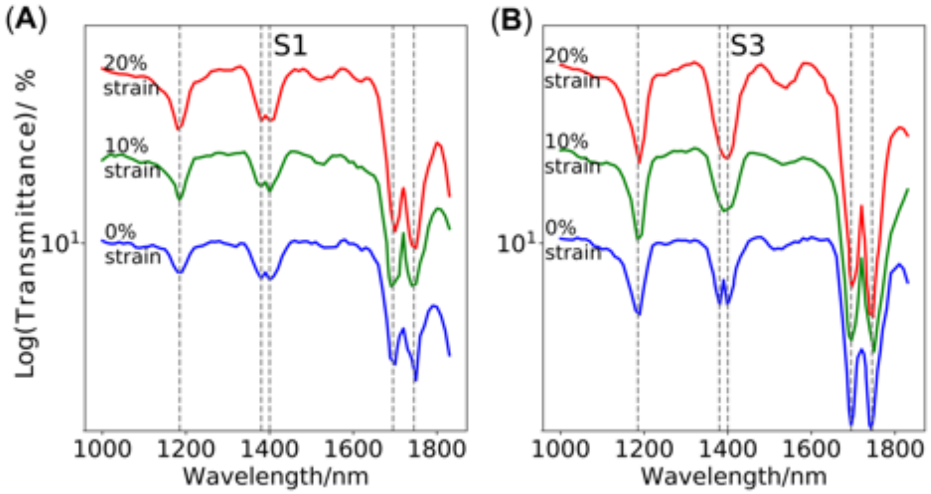

SI Figure 3. Near infrared transmittance spectroscopy measurements of S1 and S3 under no strain, 10% and 20% strain.

The behavior of the wrinkles of Ag/PDMS, under uni-axial strain, can also be examined under the NIR UV-Vis spectroscopy (**SI Figure 3A, B**), which shows significant changes with increasing strain. The absorption peaks seen at 1700-1750nm, 1400-1420nm, 1200nm are associated with the passing off of diffraction into tangentially scattered waves along the metallic surface. Electromagnetic waves scattered by the undulation of a metallic wrinkle excites bound modes known as surface plasmon polaritons (SPPs) that travel to the next undulation resulting in a collective resonance. In the absence of external strain, S3 shows a near doubling of the absorption peak intensity in all three modes, with the third order mode of 1200 nm showing a 3-fold enhancement factor. As noted in the description of the wrinkles, the increased film buckling reduces the sizes and wavelengths of the wrinkles to below that of the incident illumination wavelength. The reduction of periods leads to excitation of diffraction modes passing off at a smaller wavelength, which then could couple propagating SPPs. If the 1st order (which is strongest) is coupled, then SPP propagation distance will be greater. If higher order modes are coupled, then the SPP is weaker and hence will propagate a shorter distance. The wider Full-Width at Half Maximum (FWHM) of the peaks in S3 can also be attributed to the existence of a stronger SPP. That is, if the first order diffraction mode is coupled with the SPPs then FWHM should be smaller than if the 2nd order diffraction mode is coupled to the SPPs.

Raman Measurements

For these measurements, 1mM of DPPE-PEG in liquid phase was transferred on to the Ag/PDMS platform using Pasteur pipettes and were allowed to air dry for 30 minutes. Renishaw Raman microscopy was used for the SERS measurements. Each spectrum was corrected for cosmic rays and the background Rayleigh scattering or fluorescence emission. The following parameters were set during the SERS measurements at two wavelengths of 532 nm and 638 nm:

Wavelength	Laser power	Acquisition time	Accumulation	Objective lens
532	5 mW	10 seconds	1	N Plan 20x/0.45 NA
638	14 mW	10 seconds	1	N Plan 20x/0.45 NA

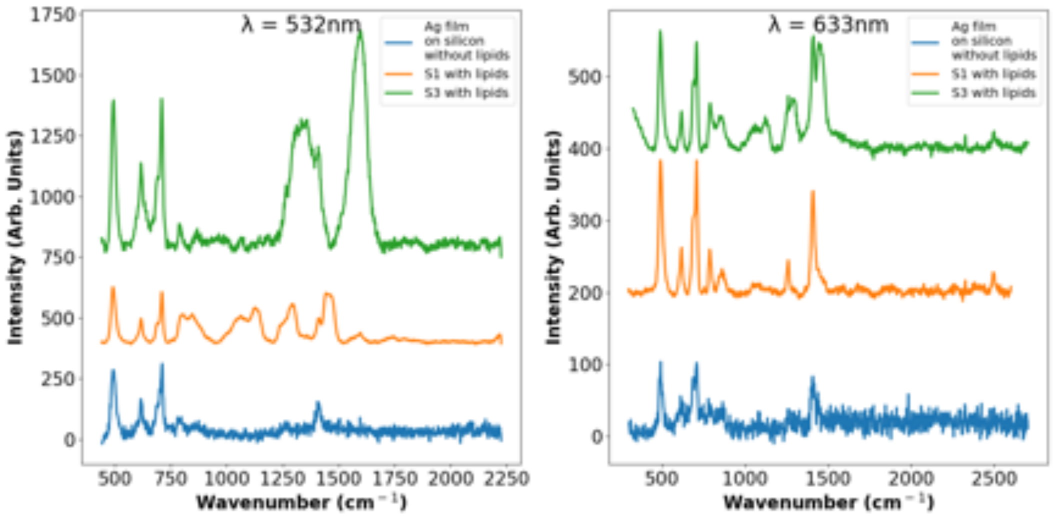

SI Figure 4. Electric field intensities at the valleys of the sinusoidal wrinkles shown in Figure 4F,G, across the width of the set of wrinkles. The field intensities are significantly greater at 532 nm incidence wavelength, correlating with the higher Raman scattering seen at 532nm in Figure 4D.

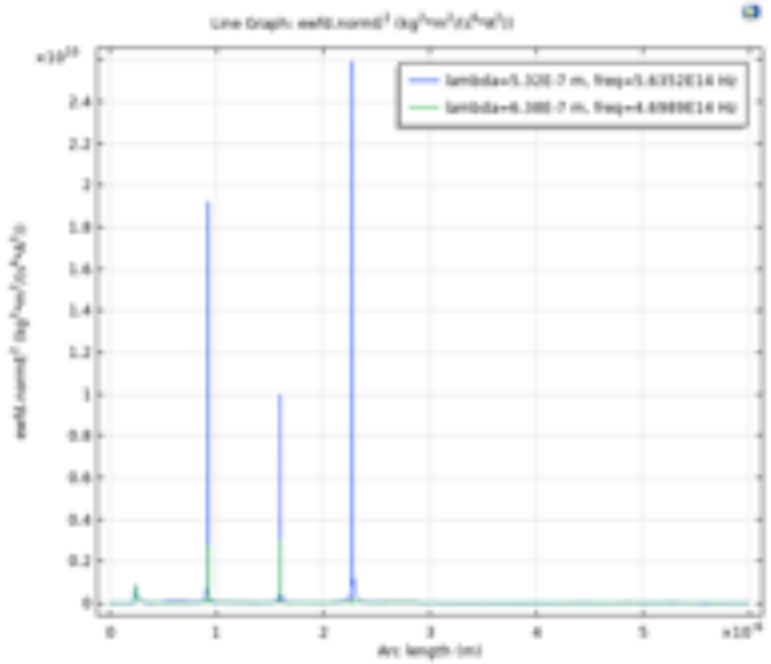

SI Figure 5. Areal averaged Raman spectrum scan over a line distance of 165 µm, taken over 330 spectra scans. A flat planar silver film is shown for comparison and the Raman peaks likely arising from intrinsic silver roughness is greatly enhanced when silver is a wrinkle. The results show that even with a beam spot smaller than the wrinkle, the averaged scan shows a significant increase of SERs in S3.

In order to compare the plasmonic response of the substrates with higher N_2 content (SI Fig 6(a)), we perform additional COMSOL simulations. We vary the wrinkles-folds period (by decreasing it in this case to mimic the effect of N2) and plot the electrical field distribution at 532 nm and 638 nm The simulations show the wrinkles in one dimension – SPP enhancement - and do not take into account the additional enhancement provided by LSP. Nevertheless, it can be seen that multiple hot spots emerge by virtue of the evanescent field superposition as the sides of the wrinkles' approach a V-like structure (SI Fig 6 (b), (c)).

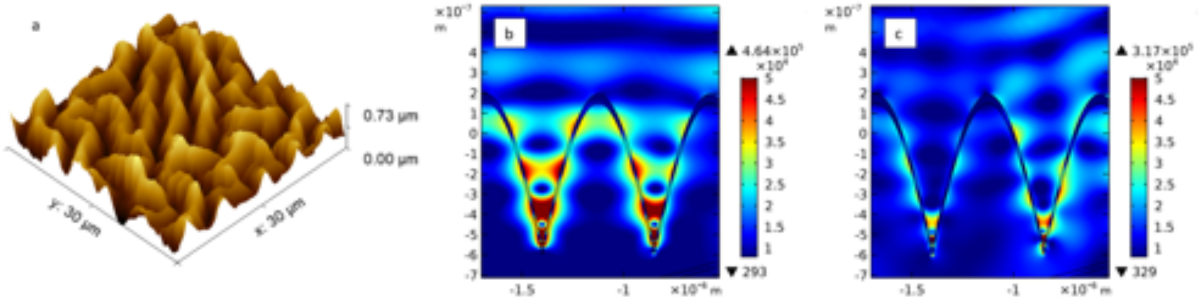

SI Figure 6. AFM surface profile of Ag/PDMS fabricated with 60% N2/Ar flow ratio (a). Electric field cross section of sharply defined valleys associated with sample under 532nm(b) and 638nm(c) incident light.

Electrical Conductivity Measurements.

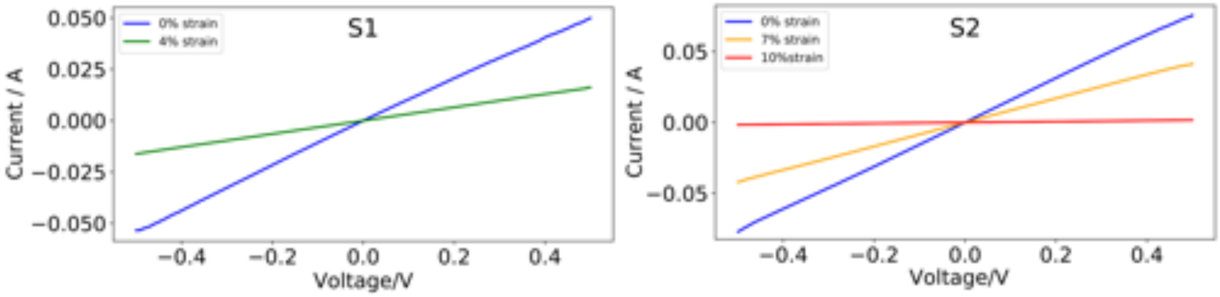

SI Figure 7. Current-voltage plots for S1 and S2 under uniaxial strain.

Custom-designed Strain-application Device.

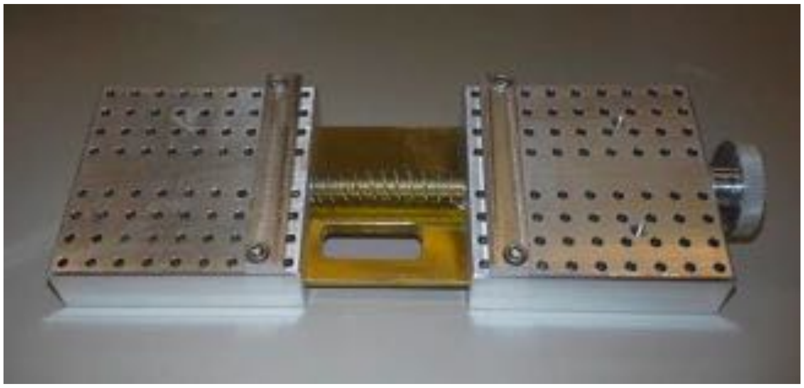

Tape Test Measurements

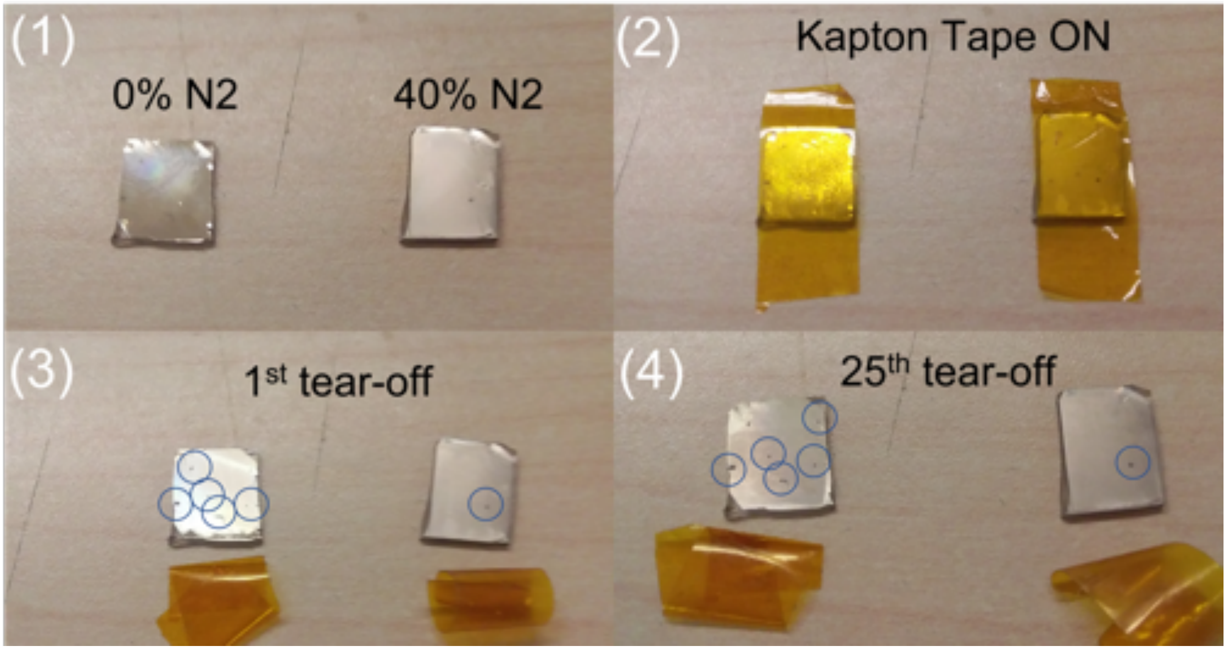

SI Figure 8. Simple tape peel-off test considering two samples: 0% N2/Ar Ag/PDMS (sample S1) and 40% N2/Ar Ag/PDMS (sample S3) which are shown prior to the application of Kapton tape in Fig. S8 (1). We note that minor defects are already visible on S1, while on S3 there are fewer defects visible. Kapton tape is applied over each sample (Fig.S8 (2)), further the tape is then firmly pressed down several times by smoothly running the finger over the tape on each sample, and then the tape is peeled off. In the first sample S1 following the tape peel-off, the initial defects are observed to have become visibly larger in size (see Fig. S8 (3)) while sample S3 is essentially unchanged. We repeat the tape tests 25 times. After 25 cycles of application and tape peel-off, no new defects are seen beyond those originally present in samples S1 and S3 albeit the initial defects have become slightly larger. Notwithstanding the initial defects, which are attributed to sample handling in a non-cleanroom environment, the silver film remains well-adhered to the PDMS layer.

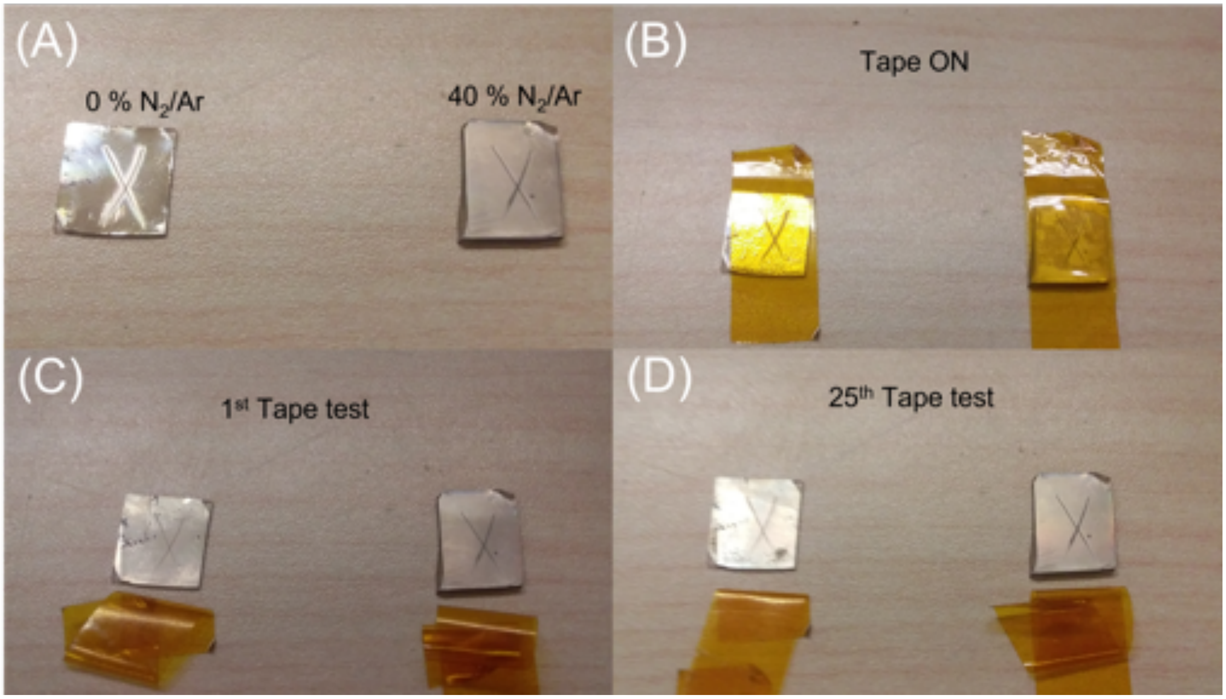

SI Figure 9. Tape test based on ASTM D3359 "Standard Test Methods for Rating Adhesion by Tape Test". In this tape test, a scalpel was used to make a cross-cut (Fig. S9 (A)), followed by application of the tape (Fig. S9 (B)) and follow-on peel off (Fig. S9 (C)); Fig. S9 (D) shows the sample following 25 cycles of the tape test. It can be seen that in the 40% N2/Ar sample the edges of the cuts are completely smooth and no visible detachment is observed. For the 0% N2/Ar sample, most of the cross-cut is preserved with small flakes detached near the intersection of the cross-cut. Based on the ASTM Adhesion Rating Scale for 'Method B', adhesion on S3 would be rated a 5B and on S1 rated a 4B (where less than 5% of the cut area is affected). According to these standards, adhesion ratings of 4 and 5 are considered acceptable in many industry applications.

References

1. Yin, J., and Chen, X. (2011) Buckling patterns of thin films on compliant substrates: The effect of plasticity. *J. Phys. D. Appl. Phys.*, **44** (4).
